# Photonic microstructures for energy-generating clear glass and net-zero energy buildings

**DOI:** 10.1038/srep31831

**Published:** 2016-08-23

**Authors:** Mikhail Vasiliev, Ramzy Alghamedi, Mohammad Nur-E-Alam, Kamal Alameh

**Affiliations:** 1 Electron Science Research Institute, School of Science, Edith Cowan University, 270 Joondalup Drive, Joondalup, WA, 6027, Australia

## Abstract

Transparent energy-harvesting windows are emerging as practical building-integrated photovoltaics (BIPV), capable of generating electricity while simultaneously reducing heating and cooling demands. By incorporating spectrally-selective diffraction gratings as light deflecting structures of high visible transparency into lamination interlayers and using improved spectrally-selective thin-film coatings, most of the visible solar radiation can be transmitted through the glass windows with minimum attenuation. At the same time, the ultraviolet (UV) and a part of incident solar infrared (IR) radiation energy are converted and/or deflected geometrically towards the panel edge for collection by CuInSe_2_ solar cells. Experimental results show power conversion efficiencies in excess of 3.04% in 10 cm × 10 cm vertically-placed clear glass panels facing direct sunlight, and up to 2.08% in 50 cm × 50 cm installation-ready framed window systems. These results confirm the emergence of a new class of solar window system ready for industrial application.

The economic opportunities and socio-economic benefits currently provided by our modern industrial civilization are contributing to rapid urbanisation growth. It is reported that 66% of the world’s population is expected to live in urban areas by 2050^1^. However, fossil fuel energy supply related concerns and environmental changes are imposing significant challenges, which must be faced by future urban areas^2^. Currently, a number of increasingly-advanced building materials capable of providing energy savings through superior thermal insulation are being developed and deployed. These “green” building materials (eg low-emissivity glass and insulated glazing systems) are, with rare exceptions, not being utilized for energy harvesting or electricity generation and play a role only in reducing the electric power consumption. In principle, integrating photovoltaic (PV) systems into “green” buildings can provide a significant additional source of energy generation located at any surface available within the building’s envelope, with the energy generated being accessible immediately at the point of use. Conventional BIPV systems integrated into building façades and rooftops can provide significant solar energy harvesting capability, which is a necessary requirement for achieving net-zero energy balance in buildings. However, providing local energy generation capacity sufficient for approaching the net-zero balance in practice still remains an ambitious goal. Achieving this goal will likely require utilization of all possible energy-generator deployment areas, including glass walls, windows, and skylights. Glass-based solar energy concentrators of high power conversion efficiency (PCE) are now expected to be deployed in next-generation windows^3^, which will enable the widespread construction of net-zero energy buildings. Earlier reports on the development of transparent solar windows date back to 2010^4^, when nanocomposite light-guide plates based on polymethyl methacrylate (PMMA) containing luminescent species have been demonstrated as viable light-harvesting components in window modules. Papers describing the various studies aimed at demonstrating new solar window designs appear in the literature^5^,^6^,^7^, however, no published sources have so far reported on the practical development of industrialization-ready highly-transparent and colour-neutral glass-based solar concentrators suitable for long-term use in building windows.

Organic luminescent materials were widely investigated during the last several decades to achieve increases in the overall power conversion efficiency of luminescent solar concentrators (LSC), because this class of photoluminophores are known to have a superior light absorption performance and fluorescence quantum yields approaching 100%. A comprehensive analysis of the performance limitations and possible efficiencies of LSC devices containing various organic dye-based materials has been reported in 1981^8^. Material development efforts aimed at eliminating the major loss mechanism (reabsorption of light emitted by luminophores) and the demonstration of transparent large-area solar window devices using nanoclustered phosphor materials with very large Stokes shift have recently been reported^9^. The recent development of semiconductor nanocrystal-based materials showing practically no overlap in their absorption and emission spectral profiles is also expected to lead towards continued rapid progress in solar window development^10^. Although some studies succeeded in reducing the disadvantages of using organic materials, the power conversion efficiencies have not yet reached the desired levels suitable for industrial applications, at least in glass-based large-area concentrators. As an example, in one study, a fluorescent organic dye was modified to selectively harvest the near-infrared light, since this is a major part of the solar spectrum along with the UV and visible spectral regions. The LSC device developed using this organic material demonstrated a very high transparency with power conversion efficiency up to 0.43%^11^. Organic dyes were also used to develop transparent near-infrared photovoltaic solar cells producing the highest power conversion efficiency obtained so far from using organic luminescent materials, at 1.71% measured in a 10 cm × 10 cm sample^12^. Although a large Stokes shift and reduced reabsorption loss were achieved in these studies, only relatively modest PCE values were demonstrated. Practical deployment-related issues in transparent organics-based concentrators or solar cells such as their environmental exposure stability have not been resolved conclusively till present. Inorganic quantum-dot-based luminescent media have been developed recently to replace conventional types of organic fluorescent materials in solar energy-harvesting applications. As an example, quantum-dot-based luminescent concentrators of sample size 12 cm × 12 cm were developed using CuInSe_x_S_(2−x)_ quantum dots embedded into photo-polymerized polylauryl methacrylate^3^. Yet, the total optical-to-electrical power conversion efficiency of the developed quantum-dot LSC device can be calculated to be about 0.576% using a Si PV cells system (if installed at concentrator edges and having 18% PV conversion efficiency under the conditions of partial geometric shading), based on the reported figure of 3.2% related to the “optical power efficiency.” In another study^7^, a thin film coating has been reported to produce a textured layer over a window that generated electrical power while maintaining 95% visible transparency. This development excluded all the disadvantages associated with using any organic fluorescent materials and the chemistry processes associated with inorganic quantum dot synthesis. One square meter of transparent window area was shown to generate electrical power sufficient for providing electro-chromic switching functionality, if an electrochromic layer was incorporated into the window design. In another recent study, researchers combined the use of nanoparticles and fluorescent dyes to increase the overall efficiency^13^. In this study, the light routing efficiency towards the edge-mounted Si solar cells was reported to be improved by using a diffractive structure formed by nanoparticles.

In order to demonstrate solar energy-harvesting window designs suitable for deployment in future buildings capable of approaching net-zero energy balance, environmentally-stable and highly transparent glass-based concentrators of higher efficiency and simultaneously providing superior thermal insulation still need to be developed. Among the recently-proposed new ideas on how to achieve substantial improvements in LSC performance by overcoming the inherent (geometry- or reabsorption-related) limitations are the use of luminescent plasmonic nanostructures containing luminophores and demonstrating directional-emission properties^14^, and even harnessing the power of stimulated emission processes assisted by using “seed laser” radiation injected into the LSC volume^15^. Virtually no research publications available to date have disclosed the development of any large-size solar concentrator-based PV windows using only inorganic luminescent materials whilst being based on clear glass without any added colouration, and generating electric power outputs on the scale of Watts, or otherwise demonstrated suitable for commercial industrialisation.

In this work, we propose a new design methodology in glass based energy concentrators, which relies on using photonic microstructures that are embedded into glass and consist essentially of a spectrally-selective (transparent) diffractive optical element, the near-surface volume regions of which are filled with luminescent media. A combination of several interrelated solar energy harvesting mechanisms plays a role in the overall performance of the developed solar concentrators of this hybrid type. We use low-emissivity thin film coatings of high visible transparency, which work in synergy with inorganic luminescent particle-loaded planar interlayers that link different parts of concentrator together and also provide luminescent disordered media redirecting the energy of incident photons towards the edge-mounted CIS solar modules^16^. Experimental results show that significant improvements in PCE are achieved in these transparent solar concentrators by employing micro-structured diffractive elements that are designed specifically to deflect only near-IR light whilst retaining high visible-range transparency and maintaining image distortion-free visual appearance. Light trapping mechanisms, such as total internal reflection (TIR), multiple scattering, diffraction and photoluminescence effects, are all being utilized in the new solar window devices in order to boost the power conversion efficiency.

## Diffractive structures for spectrally-selective light deflection

Direct geometric deflection of light rays propagating within glass-based concentrators can be provided by diffraction effects. These can be harnessed to enhance light trapping by increasing the total internal reflection probability and/or coupling the incident energy into guided modes of the structure, if either the deflection angles are sufficiently large, or if several repeated diffraction events occur for the same light ray in sequence. The main problems with using diffractive optics in window-type applications requiring clear high-transmission glass are related to the (almost inherent) image distortions, the added visual “rainbow effect” usually observed when sunlight passes through diffraction gratings, and the reduction in direct (zero-order) transmittance. Several principal hypotheses have been formulated and tested experimentally during this study. First, we proposed that it is possible to (i) design a transmission-type visually-clear diffraction grating possessing high spectral selectivity in its diffraction efficiency distribution, favouring the deflection of both the UV and near-IR photons into non-zero transmission orders, and (ii) manufacture these gratings on glass substrates of practical size (up to 100 mm × 100 mm). Second, we proposed that a transparent diffraction grating with minimised distortion of visual transmitted image (and thus having a relatively large periodicity and small 1^st^-order diffraction angle) will prove effective at maximising the luminophore absorption length, if a luminescent layer is made to practically fill the grating grooves. Third, we investigated whether the presence of scattering particles of luminescent materials inside the grating grooves could lead to greater overall light deflection efficiency. This is achieved by randomising the range of ray incidence angles onto the diffractive element structure, which in turn could broaden the range of diffracted-order angles and then also promote multiple re-diffraction events within the concentrator. All of the above-listed mechanisms could be proposed as ways of achieving improved light trapping efficiency within planar concentrators, if optimized luminophore compositions (and concentrations) with minimised re-absorption losses and suitable particle sizes are used.

**Figure 1:**
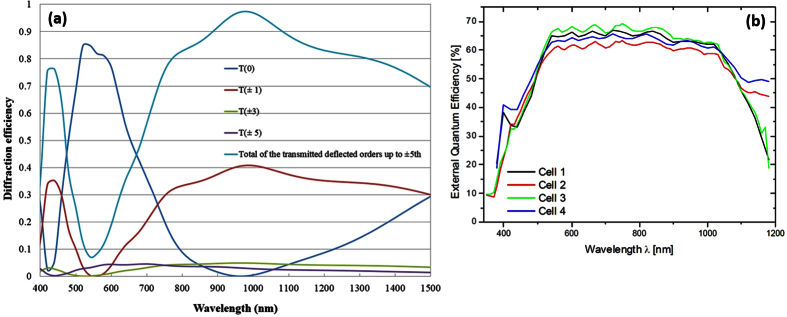
Optical properties of transparent spectrally-selective diffraction gratings and solar cells. (**a**) Modeled diffraction efficiency spectra (for unpolarised light at normal incidence) for the zero-order (direct transmission) and several odd diffraction orders of grating. The suppression of zero-order propagation for wavelengths in the near-infrared range leads to deflecting a significant fraction of the incident optical power into ±1^st^, ±3^rd^, and ±5^th^ diffracted orders, which stimulates diffuse transmission of near-infrared light; (**b**) external quantum efficiency data measured in Avancis CIS solar cells, reproduced with the authors’ permission from ref. 17.

The structure of diffraction grating suitable for preferential deflection of near-IR light was optimized using rigorous coupled-wave analysis (RCWA) software (GSolver 5.2). The principal grating design methodology was based on ensuring the zero-order suppressor functionality for wavelengths near 1000 nm and 400 nm. The core grating design idea was to maximize the amount of incident near-infrared optical power diffracted into the ±1^st^ and also higher orders, by means of interferometrically suppressing the direct transmission. Therefore, this grating design provides angular deflection, at each pass through the grating, for the incident near-IR light within the spectral region corresponding to the high-responsivity spectral band of solar cells used. Figure 1 illustrates the functionality of this spectrally-selective visibly-transparent grating in terms of re-directing near-IR energy into various diffracted orders. External quantum efficiency (EQE) data measured in solar cells from the same product family and manufacturer (Avancis GmbH, Germany) are also shown; the EQE graph is reproduced from ref. 17.

Our grating design approach focussed on patterning a thin-film coating of optimized material sequence and thickness, deposited onto a glass substrate. The grating manufacturing methodology was based on a photolithography-assisted coating lift-off process, in which a two-layer dielectric coating was evaporated onto a substrate already patterned with a developed photoresist grating. A two-layer coating design ensured flexibility to effectively optimize both the grating spectral response and also the coating adhesion properties. Another design goal was to preserve (to the extent possible) the neutral, non-colour-biased appearance of the overall glass structure developed, once the grating is embedded directly into luminophore-loaded optical epoxy interlayer. All grating design parameters have been adjusted to maximize the zero-order visible-range transmission of the unpolarised sunlight across a wide range of incidence angles. The total thickness of thin-film coating was less than 1 μm, and the gratings were produced as evenly spaced line patterns with periodicity of either 10 μm or 20 μm. We also foresaw that extending the grating design into two-dimensional (2D) space could lead to improving the performance. This is due to a more uniform distribution of light deflection directions, potentially improving the coupling efficiency of the incident sunlight into guided modes existing within the planar concentrator structures. Additional details on the grating manufacturing methodologies are reported in the Experimental Methods section.

## Glass concentrator structure, components, and materials

**Figure 2:**
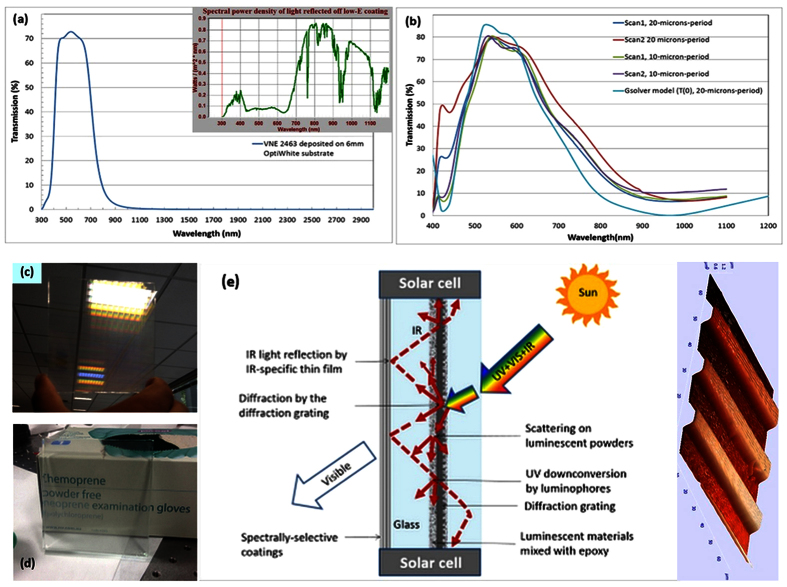
Solar energy harvesting photonic structure and its components. (**a**) Transmission spectrum of low-emissivity solar-control coating used to form spectrally-selective back-reflectors (Viracon VNE2463 deposited onto 6 mm-thick Viracon OptiWhite low-iron glass substrate); the inset shows the spectral power density distribution within solar radiation reflected off this coating, assuming normally-incident AM1.5G spectrum; (**b**) measured zero-order transmission spectra of several transparent diffraction grating structures manufactured (after being embedded into uncoated glass structure via epoxy interlayers) versus their modeled zero-order transmission; (**c**) visual appearance of a 100 mm × 100 mm, 1D, 20 μm-period diffraction grating prior to embedding into glass structure; (**d**) visual appearance of the same grating after being embedded into glass using 0.5 mm optical epoxy layer between the grating and coverglass plate; (**e**) schematic diagram (cross-sectional view) of energy-harvesting hybrid concentrators employing luminescent-media interfaces working in synergy with diffractive microstructures. An AFM image of a 20 μm-period diffraction grating manufactured using photolithography/lift-off process is also shown.

Figure 2 describes the principal features of glass concentrator design including the spectral properties of its main components (low-emissivity solar-control coating and spectrally-selective diffraction grating). It is interesting to note that the “rainbow effect” caused by the diffraction of visible light practically disappears (as was intended according to the grating design) once the grating is embedded into the glass structure using an epoxy interlayer linking the grating to a thin coverglass plate (Fig. 2(c,d)). This difference in visual appearance and transparency is caused solely by the change in the refractive index of the medium adjacent to grating structure.

The overall structure of the spectrally-selective clear-glass concentrator system consists of several components which enable it to provide energy-harvesting functionality. Low-iron glass substrate coated by high spectral selectivity solar-control coating (Viracon VNE2463) provides efficient back-reflection of incident near-IR light at all angles, as well as excellent thermal insulation properties due to low thermal emissivity. Epoxy-luminophore composite interlayers provide: multiple scattering, the luminescent down-conversion of the infrared and UV photons propagating through the structure, and the spectrally-selective diffraction grating provides an additional deflective mechanism for redirecting a fraction of the incident optical power into multiple orders of transmission and reflection. These components function together to provide a photon-management-type energy redirecting instrument for efficient incident solar power routing towards the edge-mounted CIS solar cell modules (Fig. 2(e)). The CIS modules were connected electrically in parallel and used within an optimised electrical circuit configuration containing blocking diodes and (in larger window-scale systems) also multiple bypass diodes to reduce the shading-related losses.

## Study of structure-property relationships in glass concentrators aimed at finding the clearest distortion-free designs of high energy collection performance

Multiple samples of energy-harvesting microstructured glass concentrators of size 100 mm × 100 mm were assembled, with each sample representing a structure type variation related to the type and position of the epoxy interlayer (either unmodified or functionalised by added luminophores) with respect to the diffraction grating. Table 1 describes the internal structure details relevant to each sample. All samples were characterized by way of comparing their measured electric output parameters with respect to these obtained from a reference sample employing a thin-film coating only and not containing any grating or any luminescent interlayers. The reference sample edge thickness was made equal to that of all other samples by inserting a blank BK7 glass substrate without any microstructured grating patterns into the structure, linked by two 0.5 mm clear-epoxy interlayers in-between the coated (VNE2463) 6 mm plate and 10 mm low-iron coverglass. The luminophore used was a concentration-ratio-optimized mix of two inorganic powder materials of chemistry type reported previously in ref. 16. Luminophore composition was based on Y_2_O_3_ and ZnS activated by several rare-earth ion species; the type and concentration of luminophores were identical in all functionalized interlayers. Please note that several abbreviations were used in Table 1 for brevity: REF is reference sample, LUM stands for luminophore-loaded epoxy interlayers, CLR means clear (unmodified) epoxy interlayer, and sub means substrate. More technical details on the ways in which the samples were prepared are reported within the Supplementary section.

After assembling the core structure of each concentrator sample, cut-to-size CIS solar cell modules were attached to glass edge surfaces by using the same clear UV-curable epoxy resin as the epoxy used within interlayers. The width of all CIS module stripes slightly exceeded the width of glass edges, for the following technical reasons. First, this enables the convenient addition (and also the electric reliability/serviceability) of the tabbing-wire soldering contacts after the glass structure is assembled. Second, this improves the magnitude of electric power output from samples, especially when measured under the simulated-sunlight conditions where a limited-area simulated AM1.5G beam is directed to be normally-incident onto the centre of glass, thus making it least likely for the incident photons to reach cells at sample edges. This reduced the measurement errors and magnified the sample-to-sample differences related to their structure. And third, this sample construction method allows a separate study of the relative contributions of structure related light concentration effects versus the direct (full spectrum) illumination of sunlit cell surface areas. Some details related to these direct cell illumination effects contributing to the total electric output of samples measured in outdoor conditions are considered within the Supplementary section.

Although the energy-harvesting glass design aims at achieving a somewhat uniform distribution of the optical power reaching each solar cell in the system, slightly different solar-module performances are expected as a result of incidence geometry and cell shading effects related to the positions of individual cells relative to the incoming sunlight. Therefore, special care was taken during the electric circuitry design and assembly, aiming at optimising the overall electric output by preventing cells from becoming electric loads instead of sources. The latter possibility is one of the cell shading-related effects, which is managed by using blocking diodes and minimizing the contact resistance through the use of advanced reliable soldering techniques in conjunction with chemical activators and surface cleaning.

## Sample testing methodology and energy harvesting performance comparisons

The main sample testing methodologies and related terminology have been described in our previous work on solar concentrators^16^. Indoor tests were conducted using a 2″-diameter collimated normally-incident solar simulator beam spectrally equivalent to AM1.5G solar radiation, aligned centrally with the glass of samples. The electric output parameters were then measured (Fill Factor (FF), short-circuit current I_sc_ and open-circuit voltage V_oc_) using Agilent 4156C Precision Semiconductor Parameter Analyzer for tracing the current-voltage (I-V) characteristics and a digital multimeter. The output power, the Area Collection Gain (ACG), and optical-to-electrical power conversion efficiency figures were calculated for each sample using measured data. Table 2 shows the raw data and performance parameters measured during the indoor characterisation experiments performed with a batch of twelve concentrator samples. Due to slight sample orientation-angle sensitivity of their electric output parameters and other error sources e.g. instrumentation-related inaccuracies in parameter measurements, we estimate the absolute accuracy of PCE measurements to be within ±0.005%.

**Figure 3:**
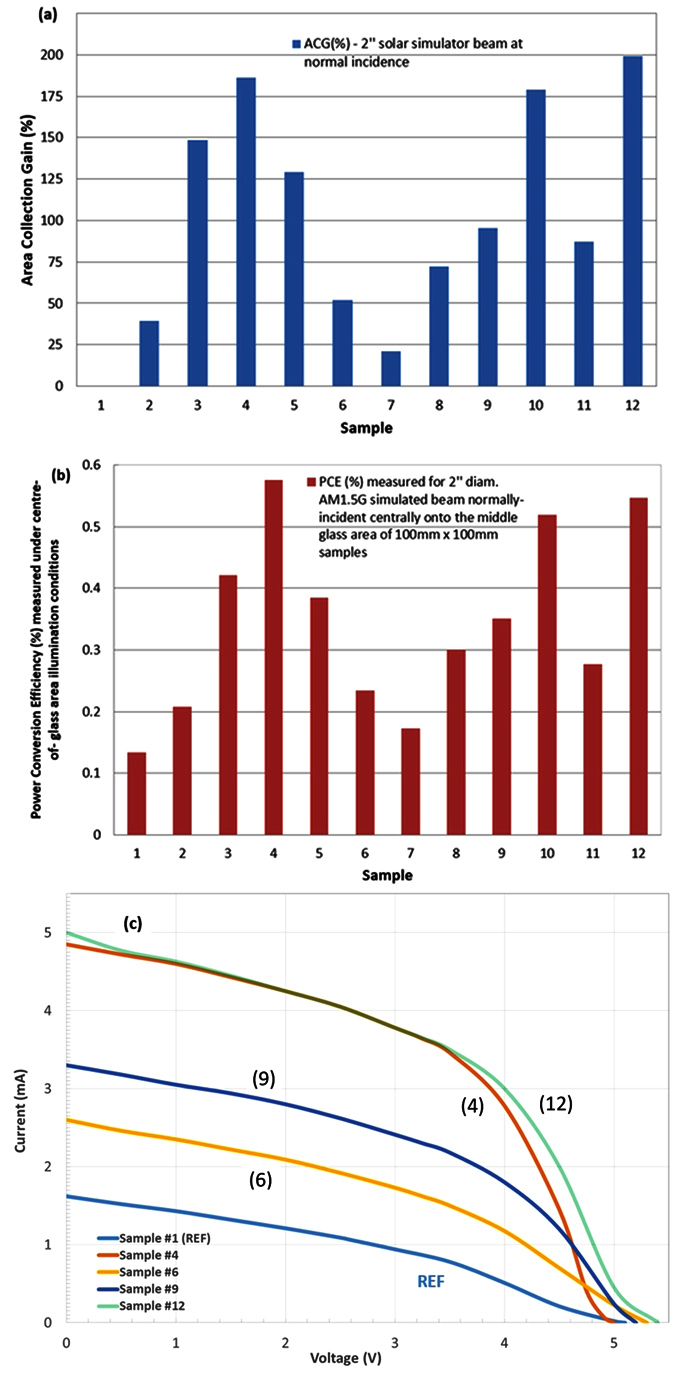
Sample-to-sample energy harvesting performance comparisons in indoor tests using collimated normally-incident 2-inch beam from a solar simulator. (**a**) Area Collection Gain (ACG) measured with respect to Sample #1; (**b**) power conversion efficiency data and comparisons; (**c**) digitized measured I-V characterisation curve traces for samples 1, 4, 6, 9, and 12.

Figure 3 provides a graphic representation of indoor sample testing results and their structure-related performance differences. It can be noted that rather substantial differences have been observed in the energy harvesting performance of concentrators. Since special care was used to ensure consistency in the sample manufacturing quality and identical component properties and assembly techniques, we can attribute these performance differences to the effects of sample structure.

It is important to note that during the indoor experiments, only the central 20.2% of total glass area was illuminated by the simulated-sunlight beam, which led to measuring only modest (up to about 0.55%) PCE values. The illuminated glass area was furthest from the solar cells, and a significant lateral distance (of several centimeters) had to be travelled by the photons within concentrators in order to reach solar cell surfaces. A very large number of simple internal reflections would have been required in this particular incidence geometry to deliver the incoming photons to solar cells and generate significant open-circuit voltages and mA-scale photocurrents. We can therefore conclude that the observed performance differences can be attributed to the structure, and that significantly larger PCE figures are expected to be measured under the real sunlight illumination conditions. We specifically selected the normal-incidence, centre-of-glass flux incidence geometry for the simulated-sunlight (indoor) tests, in order to highlight and magnify the structure-related sample-to-sample performance differences, by minimising the probability of any “random” photon collection by the solar cells.

During the outdoor tests, two main sample-testing configurations were used to characterise the energy-harvesting performance. The first was vertically-placed sun-facing position, where the Sun elevation angle could range between about 45° to 60° in different experiments, and the glass-plane normal faced the Sun azimuth direction in the horizontal plane. In this configuration, the bottom-side solar cells received the maximum amount of direct solar irradiation compared to the sides and especially the top-side solar cells. Samples placed into this position would have intercepted a maximum of 700 W/m^2^ of the total incident flux, if standard AM1.5G peak-irradiation conditions were assumed. In reality, weather-related seasonal variations in the solar irradiation intensity and spectral composition governed the actual solar flux intercepted by the samples. The weather-related solar irradiation intensity data was obtained during the experiments by using a solar irradiation meter (Daystar Meter by DayStar, Inc.), a reference calibrated CIS cell, and the local weather station data. The actually-observed short-circuit current of the reference sample also correlated closely with irradiation-related data measured. However, in all of our PCE calculations, a figure of 1000 W/m^2^ was always used for the direct-beam solar irradiation intensity, regardless of actual weather conditions, in order to provide a sufficient degree of conservatism and to remove ambiguities from the data interpretation. Since the conventional and standard practices used in the solar PV industry worldwide involve making field measurements under the real sunlight exposure conditions, using source meters or adjustable DC electronic loads, we followed the same approach and used either the PROVA 200A Solar Module Analyzer (PROVA Instruments, Inc.), or 3700 Series Programmable DC Electronic Load (Array Electronic Co., Ltd.) as measurement instruments recording the I-V curve datasets in outdoor experiments. Even though large-size (meter-scale) solar simulators could (at least in principle) be accessed, measuring the performance characteristics of large-size solar windows intended for deployment in the built environment is best accomplished in the same environment, and by using the same instruments that are in common use today in the electrical trade and solar-PV industries.

The fill factor of the vertically-oriented reference sample in near-peak flux conditions at the stable solar cell surface temperature above 40 °C and with the sample oriented in the horizontal plane to generate maximum short-circuit current was measured to be FF = 0.50, when the sample generated 150.5 mW of electric power output. The I-V curve data was recorded using PROVA 200A Solar Module Analyzer; the FF was calculated by dividing the measured maximum power output by the product of I_sc_ (43.80 mA) and V_oc_ (6.835 V). The electric parameters corresponding to the maximum power point of this sample were I_MPP_ = 34.70 mA at V_MPP_ = 4.338 V.

Sample characterisation results obtained in vertically-placed sun-facing configuration experiments conducted on September 4^th^, 2015 at midday, in weather conditions corresponding seasonally to about 80% of the peak irradiation flux are summarised in Table 3. During measurements conducted across the entire batch of concentrators, the instantaneous short-circuit current from the reference sample was tracked and recorded, to reduce or exclude the errors due to short-term weather and cloud-related incident-flux intensity variations. The main result of observations made across the batch of sun-facing samples is the clear correlation of their relative performance differences with the results obtained in indoor tests.

The second sample position used in outdoor experiments was vertically-placed peak-output-oriented position, in which the glass-plane normal was rotated in the horizontal plane by 40°–45° away from the Sun azimuth direction. At this position, the samples were intercepting less of the total incident optical power flux, about 536.2 W/m^2^ at peak conditions for a 40° sample rotation angle. The samples that were placed into this position produced greater short-circuit current for the following reasons: (i) a greater fraction of the total installed solar cells area was directly sunlit or at least not significantly shadowed geometrically, and (ii) the increased horizontal-plane flux incidence angle allowed light to reach the side-cells more effectively, after fewer internal reflections. The characterisation results obtained in peak-oriented vertically-mounted sample experiments (immediately after recording the sun-facing dataset of Table 3) are summarized in Table 4.

Again, significant structure-related performance differences were observed across the entire batch of concentrators, and similar correlations in the electric power output were observed, compared to the indoor simulator-based test results. The datasets indicate, in particular, that (i) the presence of diffraction grating on its own (without luminescent media inside structure) does not lead to any significant gains in the energy-harvesting performance; (ii) the presence of luminescent media near the diffractive element does lead to significant performance gains, clearly suggesting some “synergy” between the two energy-harvesting mechanisms, (iii) shortening the periodicity of diffraction gratings leads to better energy collection performance (which was expected, since the diffracted-order deflection angles were then larger), and (iv) there are some potential benefits in using two-dimensional grating structures in conjunction with luminescent media.

**Figure 4:**
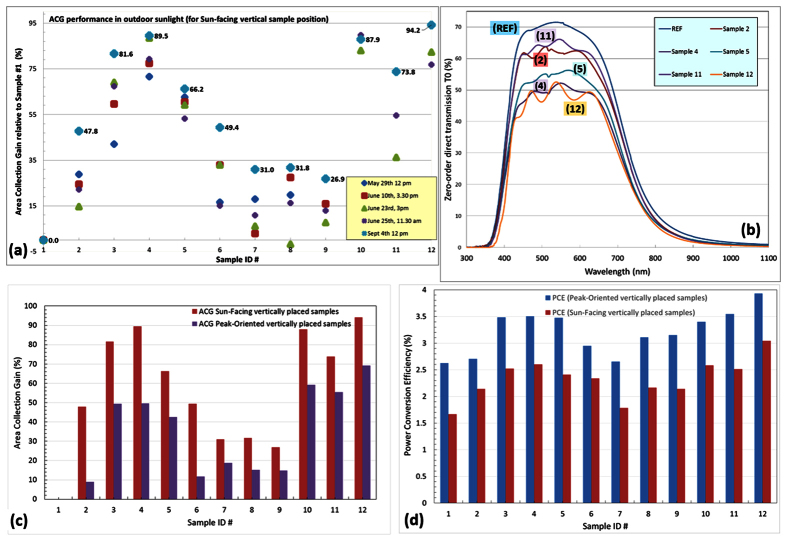
Energy harvesting performance comparisons from outdoor testing data and the optical transmission of samples. (**a**) Area Collection Gain datasets measured in outdoor tests during five measurement sessions conducted at different time- and season-dependent weather conditions; (**b**) direct (zero-order) optical transmission spectra of several samples measured using Beckman Coulter DU640B spectrophotometer; (**c**) comparison of ACG values for each sample with respect to Sample #1 obtained at sun-facing and peak-oriented vertical positions (using data from Sept 4 measurements in Perth, Western Australia); (**c**) comparisons of the total optical-to-electrical (wall-plug) power conversion efficiency across the batch of concentrators (PCE, calculated assuming standard AM1.5G irradiation conditions but using an actually-measured (Sept 4, 2015) electric output data for each sample in the sun-facing and peak-oriented vertical positions.

Multiple outdoor characterisation experiments were conducted during 2015, in order to check the consistency of results obtained in different weather conditions and at different times of day, corresponding to widely varying Sun elevation angles and actual irradiation flux intensities. Highly correlated electric output performance differences were observed across the batch in different experiments, except possibly for sample #8, which showed some unexpected variations, which could be related to the slight non-uniformity in its 2D grating pattern. Figure 4 provides a graphical illustration of the various experimental results obtained in outdoor testing experiments, including the trends observed in the “area collection gain,” the correlation of energy-harvesting performance differences with direct optical transmission of samples, and PCE for both principal sample orientations. Relatively high PCE values of up to 3.93% were demonstrated in vertically-placed samples oriented for peak output. It is also remarkable that PCE in vertical sun-facing orientation increased by a factor of 1.825 compared to the reference (unstructured and luminophore-free) sample, due to adding internal 2D microstructure and luminescent media.

## Summary of results and discussion

The effects of direct lightwave deflection provided by diffractive microstructures were shown experimentally to substantially improve the energy-harvesting efficiency of planar glass-based transparent concentrators, which also employed other light deflection mechanisms, including luminescence and multiple scattering. This was confirmed by direct measurements of electric power outputs in multiple samples performed in both the lab conditions and in multiple outdoor sunlight-illumination experiments.

In particular, adding a structure variation to the concentrator samples by embedding a transparent 1D diffraction grating alone did not improve their energy collection performance significantly, at least in indoor tests (as seen from the data related to samples 2, 6, 7, and 11). The rest of the samples, in which inorganic photoluminophore-loaded functional interlayers were employed, displayed significant energy-collection improvements compared to both the reference sample #1 and other samples. Generally, the structures that contained two functional interlayers, placed immediately above the diffractive element surface and also under the grating substrates, showed higher performances in terms of I_sc_, FF, and PCE in all testing conditions. In these samples, the photoluminescence effects and also the spreading of light caused by disorder-induced multiple scattering events were enhanced due to the increased interaction pathlengths and greater volumes of the interlayers traversed by the propagating light. Multiple diffraction-induced lightwave deflection events combined with other light harvesting mechanisms synergistically, leading to improved energy harvesting functionality, which was evident through the observed sample-to-sample correlations in light collection performance. These performance correlations were especially notable when comparing lab tests with outdoor characterisation results. Structure-related performance variations were also noticed to depend on the grating structure specifications. As was expected, using gratings of shorter periodicity and thus providing larger diffraction-order deflection angles has led to performance improvements (samples 10–12). It was also noticed that samples containing 2D grating structures formed by crossed periodic line patterns (samples 8, 9, 11, 12) demonstrated higher PCE compared to 1D grating samples (especially for samples #11 and 12). It is important to note that virtually no significant performance differences were noted when rotating either 1D or 2D samples orientation around their centre axis, since the gratings typically led to the same efficiency improvements whether diffracting the incident light toward the bottom, or the side-mounted solar cells. The results obtained confirm that using microstructured diffractive elements in conjunction with luminescent planar interlayer media within the glass structure provides a major contribution to the overall energy harvesting performance in these hybrid-type concentrator devices.

**Figure 5:**
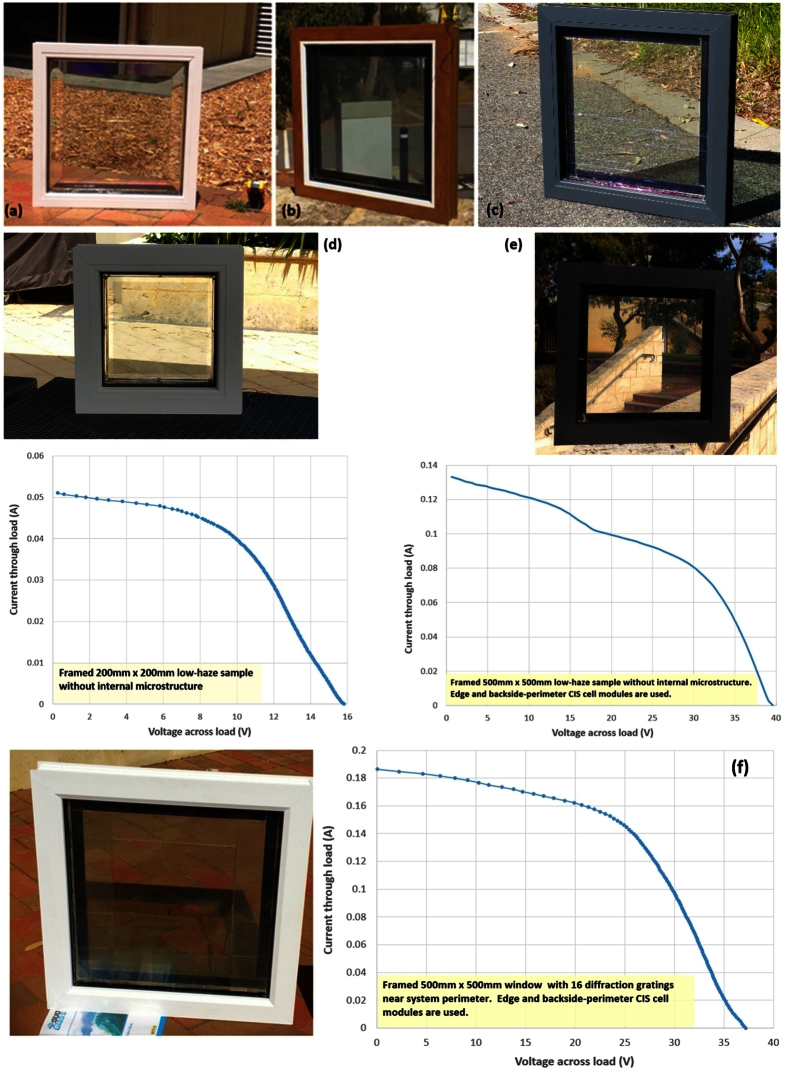
Up-scaled (glass sizes 200 mm × 200 mm and 500 mm × 500 mm) commercially-framed transparent solar concentrator windows and their electric performance characteristics. (**a–c**) Images of various framed 500 mm × 500 mm solar window systems employing only flat-glass interlayers (without using diffractive elements) and some macro-structured components for internal light deflection functionality; (**d**) framed 200 mm × 200 mm window containing a low-haze interlayer (resulting in less than 4.1% haze) and its measured I-V curve. The measured electric output parameters were P_max_ = 0.398 W and FF = 0.489; the minimum estimated PCE = 1.42%, using a 28 W figure for the maximum total irradiation flux incident onto vertical sample surface. (**e**) Framed 500 mm × 500 mm low-haze window packaged as an insulated glass unit (IGU) and its measured I-V curve. Edge-mounted and also backside-perimeter-mounted CIS solar cell modules are used to boost the energy-harvesting performance for the building industry and greenhouse applications. P_max_ = 2.431 W, FF = 0.455, and PCE = 1.39%; the maximum measured I_sc_ and V_oc_ were 148 mA and 41.2 V at the initial cell temperature near 25 °C. (**f**) Window system employing edge- and back-perimeter-mounted CIS cells, inorganic luminophore-loaded interlayer, and 16 microstructured transparent diffractive elements placed within the glass perimeter area, demonstrating electric power output of 3.64 W, corresponding to PCE of at least 2.08%. The measured fill factor was 0.525, and the maximum short-circuit current observed from this sample was 212 mA. The widths of all solar cell modules used were 27–30 mm, dependent on sample design; the solar cell surface temperatures were near 48 °C in all experiments. All characterization measurements were made at the direct-beam solar irradiation intensities not exceeding 920 W/m^2^, with window samples placed vertically into a sun-facing position.

Large-size solar window systems of several different design types were constructed with and without using embedded diffractive elements placed within near-edge glass perimeter regions. Several examples of solar windows constructed so far are documented in Fig. 5. The up-scaled industrially-framed window samples of glass panel sizes up to 500 mm × 500 mm also demonstrated the highest energy-harvesting performance when luminophore-filled embedded diffraction gratings were deployed inside their structure. More details on the characterisation results obtained with these systems will be reported elsewhere; short summaries of some PV characterisation data are presented in Fig. 5. Smaller-scale (200 mm × 200 mm) samples were also evaluated, constructed using either planar glass/interlayers, or using four separate gratings of size 100 mm × 100 mm each. The flat-glass sample of this size (containing a low-haze interlayer with a smaller luminophore concentration and IR-reflector-coated back-plate glass only) demonstrated a peak PCE of about 1.42%. Efficiency of up to 1.94% (based on I_sc_ = 73 mA, V_oc_ = 15.5 V, and FF = 0.48) was achieved in one 200 mm × 200 mm sample containing interlayers of same luminophore concentration as in smaller samples and 2D diffraction gratings of period 10 μm.

Haze (defined as diffuse transmittance fraction of the total transmittance) is an important parameter used in evaluating the performance of commercial glazing systems. Haze measurement results obtained from flat-glass (not containing any microstructured elements) regions of our energy-harvesting window panels were as follows: typically between 3.9–4.2% in systems using low-haze interlayers, and between 8–12% in systems of same luminophore concentration as used in 100 mm × 100 mm samples. Haze values well in excess of 12% would have been measured in perimeter glass regions of windows containing diffractive elements, since the gratings were designed to stimulate diffuse transmission.

It is of interest to derive an estimate for the lower limit of the “optical power routing efficiency”, defined as the ratio of the optical power received at all solar cell surfaces through the concentrator-related light propagation pathways only, to the total optical power incident onto the glass area of a vertically positioned sun-facing concentrator sample. To ensure that the effects of any direct full-spectrum illumination of the active cell surfaces on the optical efficiency estimate were completely excluded, the electric power output from the top-side, downward-facing solar cell module (not connected electrically to the other three cell modules also installed) was used. The I-V curve data of this solar cell module was measured directly (using Agilent 4156C Precision Semiconductor Parameter Analyzer), in a newer 100 mm × 100 mm sample employing Avancis PowerMax 3.5 cells of 13.3% nominal efficiency, a high-transparency diffraction grating of 30 μm periodicity, and a single luminescent interlayer inside sample structure. During the measurements conducted at midday on June 30^th^, 2016 in Joondalup, WA, Australia, the Sun altitude angle was very close to 35.0° according to the data available from online astronomy-based Sun position calculators. The maximum electric power output measured from the top-side cell module reached 36.25 mW, of which up to 10% was attributed to the effects of diffused illumination background. The measured I–V curve of this solar cell module is shown in the Supplementary section in Fig. S4(a). The maximum optical power flux intercepted by the glass area (100 cm^2^) can be evaluated using the maximum direct-beam irradiation intensity of 1000 W/m^2^ and the Sun altitude angle of 35°. The “optical power routing efficiency” of the concentrator system employing four side-mounted solar cell modules can then be evaluated as follows:


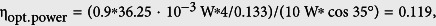


where all four CIS cell modules were estimated (very conservatively) to contribute the same electric power output as the top-side module, despite the incidence geometry considerations favouring the bottom-side solar cell areas in terms of receiving more light through internal reflections.

For the 500 mm × 500 mm windows, the diffraction gratings were installed only around the near-edge perimeter area of glass, placed near the edge-mounted CIS solar cells, since the effective propagation length of the light rays travelling inside the concentrator system towards its edges can be considered to be within 100 mm, whereas only an insignificant amount of optical power could be collected from glass centre areas. Energy harvesting improvements due to using perimeter-mounted diffraction gratings were observed clearly in samples of this size, as has been evidenced by the short-circuit current (I_sc_) readings increasing from about 140 mA (the samples of Fig. 5(b,e)) up to >210 mA (the sample of Fig. 5(f)), in similar weather conditions, and for the same open-circuit voltages of around 40 V.

## Conclusion

Highly transparent, all-inorganic photovoltaic solar window systems have been developed, which employ photonic microstructures represented by spectrally-selective transparent diffractive elements placed into direct vicinity of planar luminescent media embedded into glass structure. Experimental results have shown that the demonstrated window systems possess the potential to replace or complement conventional luminescent solar concentrator designs in building-integrated photovoltaic systems, making them suitable for use in future buildings with net-zero energy balance. Outdoor test results have demonstrated a maximum PCE of (3.041 ± 0.005)% in 10 cm × 10 cm glass-based microstructured concentrators placed vertically into the sun-facing orientation. Industrially framed solar windows of glass panel size 50 cm × 50 cm have been shown to generate up to 2.43 W (for flat-glass structures with luminescent interlayers) and up to 3.64 W of electric power output (for microstructured systems containing diffractive elements), corresponding to the power conversion efficiency of up to 2.08% measured in field conditions involving CuInSe_2_ solar modules working at their real operating cell temperature in excess of 40 °C. It has also been confirmed that the new approach to the design of glass-based solar concentrators employing diffractive microstructured layers filled with optimized luminophores and working in synergy with spectrally-selective solar-control coatings enables superior energy-harvesting performance in samples of large dimensions.

## Experimental Methods

The main components of solar energy harvesting clear glass-based concentrator structures were assembled using the methods reported in ref. 16; we also used the same methodologies as reported previously for the indoor solar simulator-based characterisation of samples. The low-emissivity thin film coatings of the solar control type used in this work have been provided by Viracon Inc. (VNE2463 deposited onto 6 mm-thick ultraclear OptiWhite glass panes with polished edges. The CIS solar PV modules glued directly onto the samples edges were rectangular-shaped manually-produced cut-outs from Avancis PowerMax modules, of nominal efficiency 12.2% and fill factor between 0.61–0.63 measured in field conditions (in manufacturer-supplied 125 W un-encapsulated modules, as well as in small solar cell cut-outs of sizes 27 mm × 98 mm and 27 mm × 498 mm, when exposed to strong sunlight). A major distinguishing feature (and unique glazing system component) used in most of the concentrator samples studied has been a visually-transparent, spectrally selective diffraction grating structure (of either 1D or a 2D near-IR deflector design type) embedded using epoxy interlayers containing inorganic luminophore particles. The structure-property relationships between the concentrator design type and their energy harvesting performance have been studied quantitatively, using both the simulated-sunlight exposure at normal incidence and outdoor electric performance measurements performed in different weather conditions.

### Transparent spectrally-selective diffraction gratings and luminescent particles

The custom-designed diffraction gratings on low-iron glass substrates have been made by micro-patterning double-layer all-dielectric coatings deposited onto glass by e-beam evaporation. Standard image-reversal photolithography process sequence has been optimised to enable large-scale (up to 100 mm × 100 mm) grating patterns formation using post-deposition lift-off approach. The photoresist type (AZ 5214E) was selected after running multiple process sequence trials using different photoresist types and layer thicknesses, to establish a production process suitable for repeatedly making uniform grating structures of minimum feature size between 5 μm and 10 μm. After running chemical cleaning processes, 100 mm × 100 mm × 2 mm glass substrates were spin-coated with photoresist using Midas System SPIN-1200D spin coater running at a maximum spin speed of up to 3000 rpm to achieve a uniform photoresist film thickness near 2 μm. This was followed by photoresist pre-exposure baking step at 110 °C for about 70 s. A Midas MDA-400M mask aligner was used in the following UV exposure step after installing the grating structure photo-mask; the photoresist-coated samples were exposed to UV light for 1 s to produce 1D grating structure. For 2D grating samples, UV exposure was applied for 0.5 s twice, using the same 1D line-patterned mask. After the first UV exposure, the sample was manually rotated by 90° on the mask aligner stage. This process yielded rather fine-quality 1D or 2D structures. Post-exposure baking step was then applied for 150 s at 150 °C to achieve reversed-image structure within the exposed photoresist layers. Flood-type (maskless) UV exposure for 10 s was then applied, and the final photoresist structures were developed using AZ 400 K diluted by 1:4 (vol.) in deionised (DI) water, by immersing the samples into developer bath for 20 s. This was followed by rinsing the samples immediately with DI water and drying in flowing nitrogen.

After developing the photoresist gratings on the samples surface, further pattern cleaning processes were applied using O_2_ plasma. O_2_ plasma ashing was conducted using Reactive Ion Etching (RIE) KVET-R2006L system supplied by Korea Vacuum Technology Ltd. Oxygen flow rate was adjusted at 12 sccm to achieve 20 mTorr of steady-state process gas pressure. Radio frequency power ignited the O_2_ plasma and was maintained at 200 W for 3–4 min ashing process duration. Plasma-cleaned photoresist gratings were then placed for thin-film deposition by e-beam evaporation using E-beam/Thermal evaporator (Korea Vacuum technology Ltd. KVE-ENT 200hl). Special care was taken during the deposition process to protect the photoresist grating structure from the excess heat resulting from the e-beam evaporative deposition process. This was achieved by providing a heat dissipation medium (heat sink) attached directly onto the back of the substrate surface inside the deposition chamber. Double-layer all-dielectric thin films were deposited onto the rotating substrates uniformly, and then the lift-off processes were run using isopropanol soaking followed by acetone immersion and DI water cleaning. Characterisation of the zero-order transmission (T0) spectra of diffraction gratings was performed using Beckman Coulter DU 640 B UV-Visible spectrophotometer, with the samples placed as far as possible from the detector slit to avoid the leakage of light from higher diffraction orders transmitted.

Lamination interlayers were formed by 0.5 mm-thick layers of transparent UV-cured optical epoxy (HK Wan Ta Shing U00049), into which photoluminescent particles of an optimized luminophore mix and concentration (of the composition type (0.025 wt%) *α* ± (0.04 wt%) *δ* (as per material descriptions first reported in ref. 16) have been added (in some interlayers only, as per samples description data of Table 1). The measured absolute fluorescence quantum yield (QY) of luminophore *α* was (4 ± 0.2)% for emissions in the range between 927 and 1150 nm excited by 905 nm light. This figure exceeds the QY values of several modern organic dye-based luminophores capable of infrared excitation reported in ref. 18. The near-infrared excitation wavelength range of this luminophore (reported in ref. 16) is remarkably broad spectrally, extending from below 900 nm to over 1000 nm. Particles of this Y_2_O_3_-based material had an average size of several μm, and their distribution within interlayers led to diffraction halo formation and easily observable light spreading effects when illuminated by visible laser sources. The fluorescence quantum yield of Ag-activated ZnS-based luminophore (*δ*) was reported in ref. 19 to exceed 40%. The epoxy-luminophore nanocomposite-type functionalised interlayer materials were directly filling the grooves of diffraction gratings in all samples using this structure type. Additional technical details on the ways in which the samples were constructed as well as the data related to fluorescence quantum yield measurements have been reported in the Supplementary section.

### Device characterization procedures

The main technical and procedural details describing the lab-based characterisation of concentrator samples have also been reported in ref. 16. During the outdoor tests, all samples have been exposed to real sunlight illumination conditions during periods of relatively stable irradiation intensity. The electric output data and parameters measured were the I-V curve datasets, fill factor, the open-circuit voltage V_oc_ and the short-circuit current (I_sc_) generated by each sample. The measured variations in the simultaneous electric output (I_sc_) of any reference samples used during the overall-batch measurement time-frame (about 10 minutes) were taken into account, to exclude any unexpected irradiation-intensity-related errors. Special care was taken to ensure that all samples have been exposed to sunlight for a sufficiently long period of time to ensure the uniformity of solar cell temperature increases across all samples under test. Two principal testing-geometry configurations were used (vertically-placed sun-facing and vertically-placed peak-output-oriented), as explained above. During all outdoor measurement sessions recorded, the Sun elevation angle exceeded 45°; nevertheless a figure of 700 W/m^2^ was used consistently for the solar irradiation flux intensity falling onto vertically-oriented glass surfaces for the purpose of making minimum efficiency estimates, regardless of the actual weather conditions. Depending on the actual Sun elevation angle and seasonal weather-related variations in solar irradiation intensity and its spectral distribution, significant variations in the relative electric-output performance of differently-structured samples were also observed in various alternative configurations of sample placement (e.g., for the horizontal placement, or for the samples tilted slightly off the horizontal position), which often exceeded the relative output-power differences observed in standard testing configurations. These observations warrant the necessity for further refining the outdoor testing procedures to devise a “concentrator performance function” which would meaningfully quantify the yearly-average energy-harvesting output obtainable from concentrator windows of any given design type and placed into all possible architectural deployment configurations. This will require further research and processing large volumes of data from multiple measurements obtained in different weather conditions, sample placement orientations and irradiation geometries. The data collected from various concentrator design types so far does suggest that for each two samples differing by a factor of two in their electric output power as measured in lab tests (using normally-incident solar simulator beam), an “orientation-averaged performance difference” of 30–40% is seen in outdoor testing, after considering all data obtained at several sample placement geometries with respect to the Sun elevation angle and Sun azimuth direction.

## Additional Information

**How to cite this article**: Vasiliev, M. *et al*. Photonic microstructures for energy-generating clear glass and net-zero energy buildings. *Sci. Rep.* **6**, 31831; doi: 10.1038/srep31831 (2016).

## Supplementary Material

Supplementary Information

## Figures and Tables

**Table 1:** Samples structure variation details.

Sample ID	Structure type variations
1	REF (Low-E coating only, no luminophores, no gratings). Two 0.5 mm clear epoxy interlayers used to incorporate a flat 2 mm-thick BK7 substrate identical to all grating substrates
2	2-layer dielectric grating on a 2 mm BK7 substrate; 1D lines; period 20 μm; clear epoxy + clear epoxy interlayers (no luminophores)
3	2-layer dielectric grating, 1D, period 20 μm. LUM epoxy layer under the grating sub; CLR epoxy layer over the grating lines
4	2-layer dielectric grating, 1D, period 20 μm. LUM under grating sub, LUM over the grating lines
5	2-layer dielectric grating, 1D, period 20 μm. CLR under grating sub, LUM over the grating lines
6	2-layer dielectric grating, 1D, period 10 μm. CLR under grating sub, CLR over the grating lines
7	2-layer dielectric grating, 2D, period 20 μm. CLR under grating sub, CLR over the grating lines
8	2-layer dielectric grating, 2D, period 20 μm. LUM under grating sub, LUM over the grating lines
9	2-layer dielectric grating, 2D, period 20 μm (design #2 marginally different from standard grating design in terms of material layer thickness). LUM under grating sub, LUM over the grating lines
10	2-layer dielectric grating, 1D, period 10 μm. LUM under grating sub; LUM over the grating lines
11	2-layer dielectric grating, 2D, period 10 μm. CLR + CLR
12	2-layer dielectric grating, 2D, period 10 μm. LUM + LUM

**Table 2:** Solar simulator testing dataset and sample testing results.

Sample	Electric Output	(averaged over two axially-rotated sample orientations)		Vertically-placed samples at 100 mm from source aperture (2″ diam. simulated AM1.5G beam incident normally and centrally)	
	I_sc_	V_oc_	(I_sc_*V_oc_)	Measured FF/PCE (%)	ACG (%)
	mA	V	mW	100%*(I_sc_*V_oc_*FF/P_inc opt_)	100%*(I_sc_*V_oc_ − I_sc Ref_*V_oc Ref_)/(I_sc Ref_ *V_oc Ref_)
1	1.660	4.920	8.167	0.330/0.133	0
2	2.410	4.730	11.399	0.368/0.207	39.6
3	3.780	5.370	20.298	0.420/0.421	148.5
4	4.440	5.265	23.376	0.499/0.575	186.2
5	3.660	5.110	18.702	0.417/0.385	129
6	2.385	5.205	12.414	0.382/0.234	52.0
7	1.995	4.960	9.895	0.353/0.172	21.2
8	2.715	5.185	14.077	0.434/0.301	72.4
9	2.985	5.350	15.970	0.445/0.351	95.5
10	4.335	5.255	22.780	0.462/0.519	178.9
11	2.985	5.115	15.268	0.366/0.276	86.9
12	4.465	5.470	24.424	0.454/0.547	199

**Table 3:** Outdoor testing results obtained with sun-facing vertically-placed samples.

Sample	Electric Output Parameters				
	Sun-Facing Vertically-Placed Samples				
	I_sc_	V_oc_	REF I_sc_	FF/PCE (%)	ACG (%)
	mA	V	mA	Estimated minimum PCE data are calculated using the measured I_sc_, V_oc_, and FF produced by each sample in field conditions. The figure used for the total solar irradiance flux intercepted is 7.0 W. The dataset was recorded at the (direct-beam) irradiation intensity conditions of <900 W/m^2^.	
1	33.0	6.27	33.0 (max. 37.2)	0.50/1.666	0
2	43.5	7.03	33.0	0.49/2.141	47.80
3	53.0	7.09	33.0	0.47/2.523	81.61
4	54.5	7.26	33.3	0.46/2.600	89.51
5	46.9	7.49	33.7	0.48/2.409	66.25
6	42.7	7.37	33.6	0.52/2.338	49.38
7	38.6	7.35	34.6	0.44/1.783	30.97
8	39.0	7.33	34.6	0.53/2.164	31.77
9	38.4	7.23	34.9	0.54/2.142	26.88
10	57.0	7.38	35.7	0.43/2.584	87.93
11	52.6	7.27	35.1	0.46/2.513	73.76
12	58.6	7.73	37.2	0.47/3.041	94.21

**Table 4:** Outdoor testing results obtained with samples placed vertically but oriented for peak I_sc_ output.

Electric Output Parameters					
Peak-Current Oriented Vertically-Positioned Samples					
Sample	I_sc_	V_oc_	REF I_sc_	FF/PCE (%)	ACG (%)
	mA	V	mA	Estimated min. PCE data are calculated using the measured I_sc_, V_oc_, and FF produced by each sample in field conditions. The max. figure used for the total solar irradiance flux intercepted is (7.0 W * cos 40°) = 5.362 W, to account for the exposure geometry details. The dataset was recorded at the (direct-beam) irradiation intensity conditions of <900 W/m^2^.	
1	40.20	7.00	40.2	0.50/2.624	0
2	44.50	6.65	38.8	0.49/2.704	13.83
3	58.70	6.77	38.0	0.47/3.483	56.09
4	59.20	6.90	39.0	0.46/3.504	56.33
5	54.90	7.07	38.9	0.48/3.475	48.92
6	42.95	7.08	38.9	0.52/2.949	16.67
7	45.80	7.06	38.9	0.44/2.653	24.06
8	45.10	6.97	39.0	0.53/3.107	20.30
9	45.20	6.92	38.9	0.54/3.150	20.01
10	60.10	7.05	38.0	0.43/3.398	66.42
11	60.00	6.89	38.0	0.46/3.546	62.37
12	61.30	7.32	37.9	0.47/3.933	76.71
