# Supplementary material for: Photonic microstructures for energy-generating clear glass and net-zero energy buildings
Source: Sci Rep. 2016 Aug 23;6:31831. doi: 10.1038/srep31831 (PMC4994116; doi:10.1038/srep31831)
Supplement: Supplementary Information [file srep31831-s1.doc]

**Supporting Online Material for Photonic microstructures for energy-generating clear glass and net-zero energy buildings**

**Mikhail Vasiliev*, Ramzy Alghamedi, Mohammad Nur-E-Alam and Kamal Alameh**

*Electron Science Research Institute, School of Science, Edith Cowan University, 270 Joondalup Drive, Joondalup, WA, 6027, Australia*

*Corresponding author. E-mail: m.vasiliev@ecu.edu.au

**Sample assembly processes and structure-related technical details**

A schematic diagram detailing the internal structure of typical microstructured concentrator prototypes is shown in Figure S1. All concentrators within our 12-sample batch except the reference Sample #1 contained diffractive elements (1D or 2D gratings of periodicity either 10 μm or 20 μm) formed by lift-off lithography patterning of double-layer dielectric thin films of total thickness less than 1 μm. Sample #1 contained only a blank 2mm-thick flat BK7 substrate surrounded by luminophore-free 0.5mm epoxy layers on each side. All samples contained a low-emissivity solar-control coating provided by Viracon, Inc. (Minnesota, USA) applied to the back surface of each composite glazing system. CuInSe_2_ (CIS) solar cell module cut-outs of size 98mm × 27mm (Avancis PowerMax) were glued directly onto flat edges of glass using a UV-curable epoxy; electrical connections to tabbing wires were soldered onto CIS cell surfaces, and all four cell modules were connected electrically in parallel using one blocking diode per module. Avancis GmbH CIS modules of nominal efficiency 12.2% have been used for the construction of 100mm × 100mm samples, and CIS modules of 13.3% efficiency (also made by Avancis GmbH) have been used in up-scaled concentrators with glass area size 500mm × 500mm.


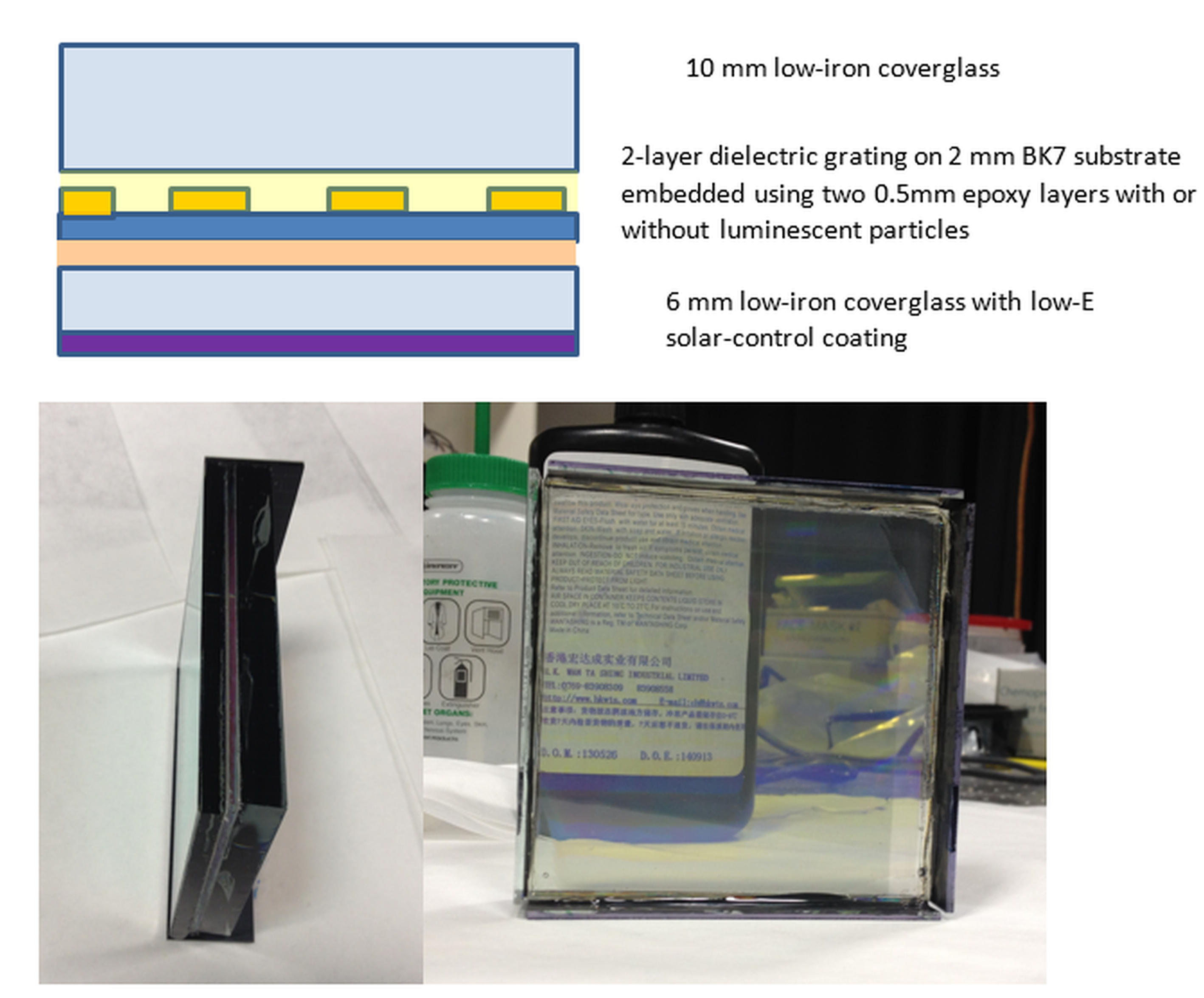


**Figure S1.** **Structure of samples.** Cross-sectional diagram of a typical microstructured concentrator sample and photographs illustrating the construction and visual appearance of 100mm×100mm concentrators.

**Outdoor sample testing methodologies**

The main electric output parameters of concentrator samples (short-circuit current I_sc_, open-circuit voltage V_oc_, and FF) were measured under real sunlight illumination conditions at different (weather- and season-dependent) irradiation conditions. The outdoor measurements were normally conducted on clear sunny days, when the measured direct-beam solar radiation intensity exceeded 750 W/m^2^. The measured electric output parameters, in conjunction with the I-V curve measurement data obtained using either PROVA 200A Solar Module Analyzer or a Programmable DC Electronic Load system yielded the module fill factor of parallel-connected CIS cell-module circuits installed into samples. Due to the combined effects of cell surface temperature increases and non-uniform geometric shading of solar cell surfaces facing the glass edge areas, the FF measured in glass window samples was significantly lower than its published nominal value (the measured FF was typically between 0.48-0.52). The electric characterisation data obtained were sufficient for making comparisons of the relative energy-harvesting performance of all samples with respect to the reference (Sample #1). Since our main objective was to study the relative performance differences between numerous concentrator samples of different internal structure, we only derived the estimates for the peak-performance power conversion efficiency of our samples. These estimates were calculated conservatively from the measured data, using a 1000 W/m^2^ figure for the direct-beam solar irradiation intensity, regardless of the actual weather conditions during the measurements. The intended application area of this research (buildings with net-zero energy consumption and agricultural greenhouses) requires optimization of the kWh/year/m^2^ energy-generation figures, as well as the yearly-average and orientation-average power output parameters of future solar windows. More research is still required to characterise comprehensively the actual energy-generating potential of diffraction-assisted solar windows. As expected, the concentrators employing optimized combinations of diffractive elements and luminescent layers demonstrated an increased stability of their power output with changes in the horizontal-plane orientation angle. This is evident from the data of Figure 3 in the main manuscript, where the power conversion efficiencies are presented for each sample type and two substantially different physical orientations. Figure S2 illustrates the outdoor testing of samples and showcases the significant performance differences between samples of very similar visual appearance, but constructed using different components.


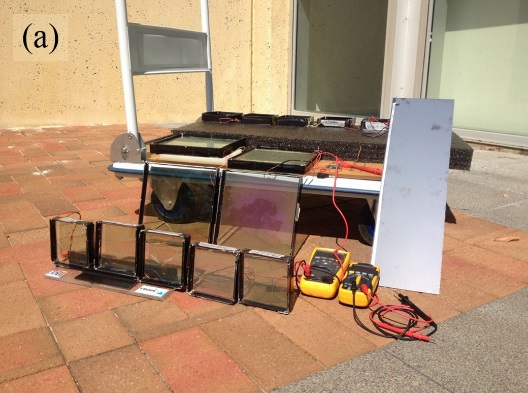

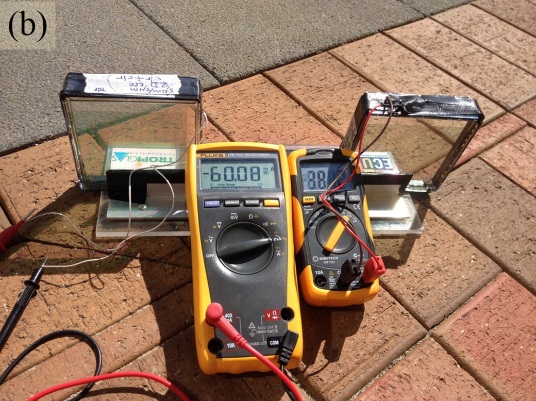


**Figure S2.** **Outdoor tests.** (a) Batch of 100mm × 100mm and larger samples during performance characterization experiments; a large cut-out of CIS cell module of known area used as reference for solar irradiation intensity measurements, in addition to using a reference sample and weather station data; (b) outdoor measurements - Sample #11(left) next to Sample #1 (right) showing a substantial I_sc_ performance difference in identical conditions (60 mA vs 38 mA).

**Role of structure-dependent flux concentration versus the effects of direct full-spectrum illumination of the exposed PV module areas**

It is important to provide a quantitative estimate (or even a back-of-envelope calculation) for the relative contribution of the directly-exposed PV cell areas, to the total electric power output generated by the concentrator structure. Vertically standing 100mm × 100mm glass samples using 27mm × 98 mm CIS cell modules glued to the edges of concentrator were used, with the glass being rotated horizontally to achieve ≈ 40° Sun azimuth angle with respect to the glass-plane normal. Based on the geometry of concentrator exposure to the incoming sunlight at peak power-output conditions, it is easy to provide an estimate of the contribution of the two directly-exposed PV cell surfaces (the bottom cell and one of the side-cells), to the total measured short-circuit current at peak illumination conditions. It is more difficult to accurately quantify the detrimental effects of partial geometric shading near the corner regions of sample imposed by the overhanging top cell module and the adjacent shaded side-cell modules, on the total electric output. Therefore, these shading effects will be ignored in our simplified calculation. The small contribution of non-concentrator-related (diffused illumination background) light reaching the geometrically-shaded side-cell and also top cell will also be ignored. Electric power loss that occurs because of ohmic losses due to small parasitic resistance in the soldering contacts will not be considered. For a Schottky diode-related voltage drop value of at least 0.7 V and an average current generated by each of the four PV modules of around 15 mA, the total blocking diode-related electric power losses are around 40 mW. The blocking diodes are therefore dissipating well in excess of 15% of the total electric power generated by any 100mm × 100mm samples. However, it is still necessary to use them, to prevent more significant power losses, which otherwise would occur due to the intrinsically non-uniform distribution of shaded cell areas and illumination intensities across the four parallel-connected solar cell modules installed in each sample.

A schematic diagram explaining the solar cell exposure geometry, and the effect of refraction on the effective solar module cross-section width calculations for the case of 45° vertical-plane incidence angle is shown in Figure S3.


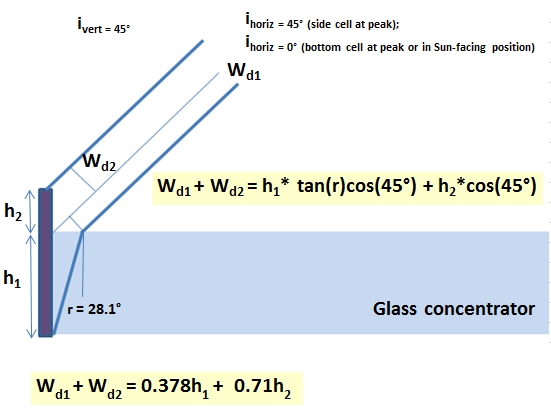


**Figure S3.** **Direct-flux incidence geometry.** Exposure of solar cell modules to direct full-spectrum flux, and effective direct-flux cross-section width calculations accounting for the geometric features of concentrator assembly.

Direct-beam total (direct and diffused) AM1.5G spectrum at peak illumination conditions characterized by 1000W/m^2^ of peak irradiation intensity will be used for the calculation of the sunlit horizontal cell module output, using the effective direct-beam exposure width of W_d1_ + W_d2_ = 12.86 mm (for h_1_ = 19 mm and h_2_ = 8 mm). The calculation of peak-orientation direct-flux electric output from a vertically-positioned sunlit side-cell involves using two additional angles (the vertical and horizontal flux incidence angles of ≈ 45° and ≈40°), which will reduce the incident direct-flux irradiation intensity over that cell module to I_side-cell vert_ = 1000W/m^2^*cos45°*cos40° = 541.7 W/m^2^. In order to account for the effects of incident flux back-reflection off the glass, we reduced the incident irradiation intensities to 95% of their values, resulting in the bottom-cell irradiation of I_bottom-cell_ = 950 W/m^2^ (within direct beam), and I_side-cell vert_ = 514 W/m^2^.

Without assuming any (somewhat inevitable) PV conversion efficiency losses in the rated efficiency of solar cell modules (12%) due to cutting, handling, and electrically interfacing small-area CIS module cut-outs, and not accounting for the effective cell area loss due to soldering tabbing-wire contacts at each end, we can now approximately estimate the expected electric output parameters of the two directly-sunlit solar cell modules at 25°C surface temperature, in the following way:

P_elec. direct-flux_ = P_bottom cell_ + P _side-cell_ = A_eff_ * η * (I_bottom-cell_ + I_side-cell vert_) = 221 mW.

This figure neglects any scattering, or diffused-transmission, or light-redirection related losses occurring within the near-edge regions of glass concentrator. Taking into account the (i) power generation loss due to the temperature coefficient of power (-0.39%/°C), and the difference between 25°C and the actual operating cell temperature (min. 48°C) when measured at peak outdoor illumination conditions, and (ii) estimated 40 mW of electric power loss across the four blocking diodes, we get:

P_elec. direct-flux @ 48C_ = 161.2 mW. Therefore, the estimated maximum short-circuit current output is:

I_sc_ _direct-flux_ = P_elec. direct-flux_ / (FF * V_oc_) = 32.9 mA,

where we used an FF = 0.62 (since this is the FF measured typically in Avancis cell module cut-outs of 12% efficiency heated up to 48-50°C when illuminated by the direct outdoor sunlight), and V_oc_ = 7.9 V measured typically in fully-exposed connectorized cell cut-outs at the real operating temperature, before the blocking-diode connections are made. In reality, accounting for the additional ohmic losses at soldering contacts would have reduced this estimated short-circuit current further, to figures below 30 mA. Any further reductions in the overall module fill factor linked to the reductions in overall module efficiency, expected to occur in a set of four parallel-connected CIS modules due to non-uniform geometric shading, are not factors which could possibly increase the I_sc_ over this estimated maximum 32.9 mA value. The experimentally measured fill factors of ≈0.5 in 100mm×100mm samples subjected to strong sunlight illumination confirm that the non-uniform shading distribution across the active cell areas leads to additional electrical mismatch effects that affect the overall module circuit operation and causes some reduction in the effective cell-area efficiency to figures well below 12%.

Despite the observed effects of cell shading on the fill factor and efficiency, the short-circuit currents actually measured so far in the reference 100mm×100mm samples (Sample #1, and also in several other structures of identical design) in summer-time peak outdoor illumination conditions were 50.5mA and 45 mA for the cases of peak-output-oriented and sun-facing vertical sample orientations, accordingly. This means that up to 53% in short-circuit current output was gained due to using the simplest of coated-glass concentrator structures, compared to the calculated I_sc_ generated simply due to the direct full-spectrum exposure of sunlit cells. Peak-weather I_sc_ outputs from some of the 200mm×200mm structured concentrators employing luminophores and diffraction gratings, which have so far been measured, exceeded 75 mA. Figure S4 shows the measured I-V curves and the corresponding power-voltage curves for several 100mm×100mm samples and illustrates the gain in the electric power output of about 40% achieved due to adding microstructured diffractive elements and luminophores to glass concentrator structure. The fill factor of reference sample varied between 0.48 and 0.52 in different measurements due to low-light effects affecting the CIS cell performance, and an average FF of 0.50 was used in all calculations relevant to this sample.


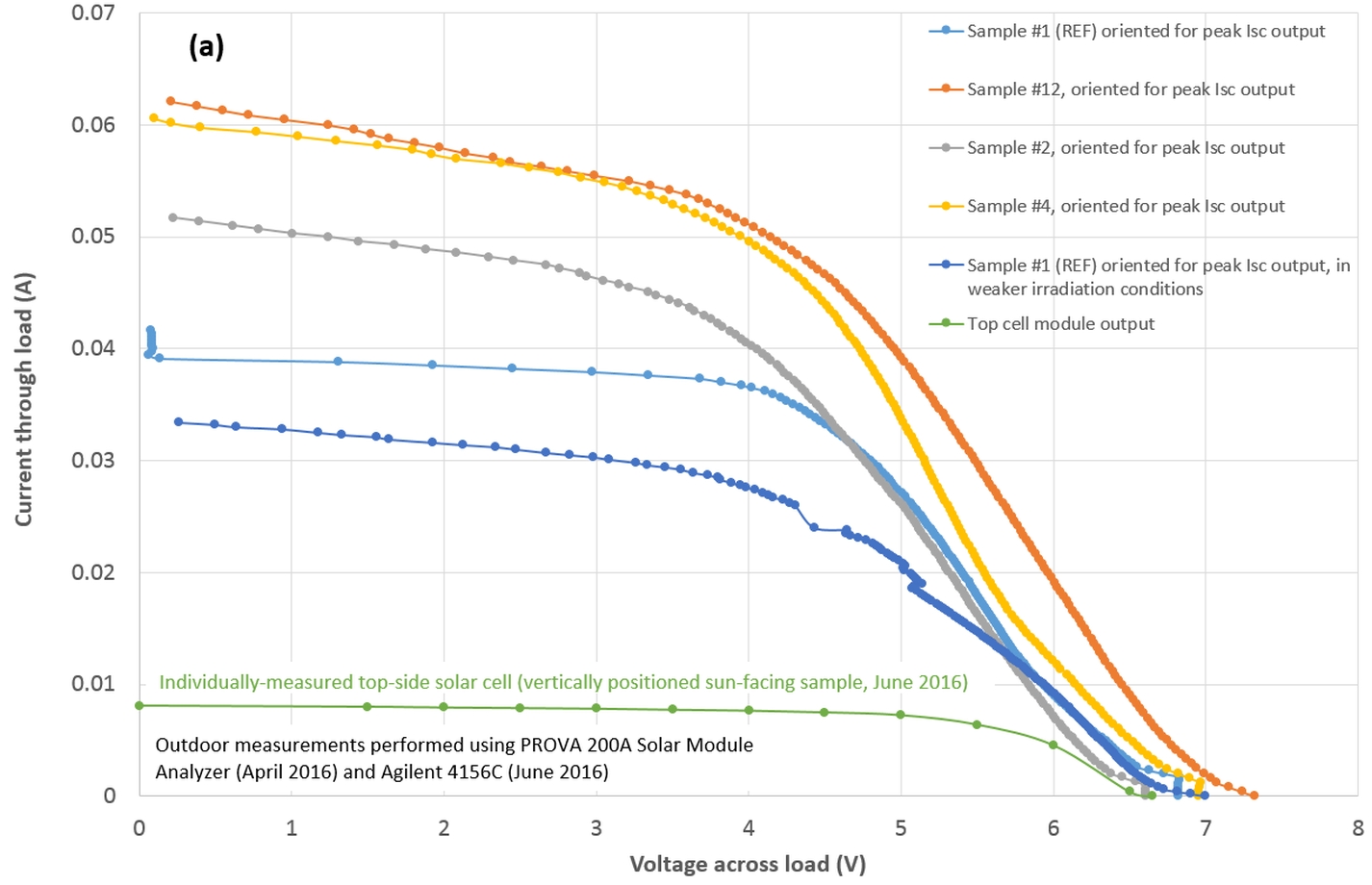


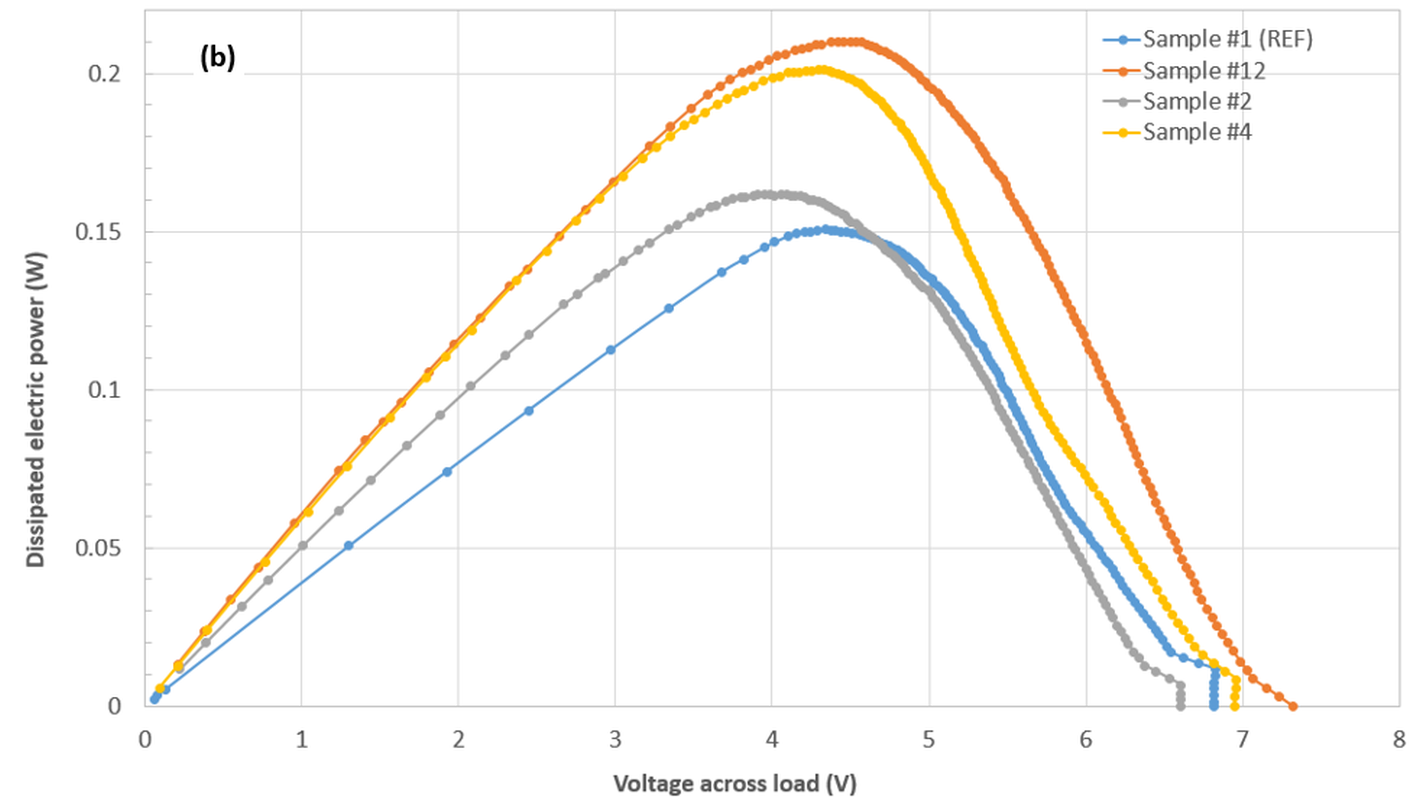


**Figure S4.** **Measured** **I-V curve data and power-voltage curves of several 100mm×100mm samples.** (a) I-V curve datasets of several vertically-positioned concentrator samples recorded in identical autumn weather conditions (April 2016), and the separately-measured I-V curve of a top-side solar cell module installed into an additional sample measured in June 2016. The bottom trace of sample #1 was recorded at weaker illumination conditions (on a different day); (b) the power-voltage curves data.

Measurements performed in outdoor experiments with a modified reference sample structure, in which the top-side solar cell module was replaced by a mirror surface of the same geometric dimensions as solar cell cut-out have revealed that (i) module FF dropped to around 0.3, and (ii) the short-circuit current was reduced by 8-10 mA. This further illustrates the energy-generation contribution of the top-side solar cell, the active area of which is facing downward and receives no direct sunlight.

**Theoretical performance limit estimates for the electric power generation in highly-transparent spectrally-selective concentrators**

It is interesting to estimate the theory limits for the maximum possible amount of the optical power distributed across all parts of the incident solar spectrum within the resposivity bandwidth of CIS cells which could, in principle, be delivered towards the edges of the 100mm×100mm concentrator structure at peak illumination conditions. Calculations will be made for sun-facing vertical sample positions, assuming 100% light-routing efficiency and total absence of any loss mechanisms. Tropiglas Spectral Calculator software (developed by M. Vasiliev at ECU) can be used to integrate the incident AM1.5G power density distribution across the spectral regions of interest, whilst accounting accurately for the spectral filtering effects of the solar-control coating applied to the back of glass structure. The calculation results (at a glance) can be summarized in the following way:

For a vertically-placed sample having its glass-plane normal aligned in the horizontal plane with the Sun azimuth angle, at 45° Sun zenith angle, the incident peak-weather solar irradiation flux intercepted by the concentrator area of 0.01 m^2^, is P_flux, total_ = 0.01 m^2^ * 1000 W/m^2^ * cos (45°) = 7.07 W. Using Tropiglas software and accounting for the 5% of front reflection losses and the exact spectral properties of low-emissivity coating, the total optical power between 300 nm and 1250 nm transmitted through the concentrator will then equal about 2.96 W. This leaves, theoretically, up to 3.71 W of the total incident optical power available for re-direction towards the edge-mounted solar cells. If the light re-direction efficiency was 100% over the entire glass area, the maximum electric power output harvested by the 12.2%-efficiency CIS cells would have been only 0.453 W.

In the sun-facing vertically-oriented sample position, only the bottom solar cell receives the direct full-spectrum sunlight illumination, and therefore only about 105 mW of this direct-light power contribution needs to be added to the calculated theoretical figure. We can then derive the theory-limit for the maximum electric power output possible from a 100mm×100mm vertically-placed sun-facing concentrator sample being ≈ 0.558 W. This would correspond to a theoretical maximum PCE of ≈ 7.89%.

The calculated results also confirm that strong energy harvesting performance was achieved in microstructured concentrator samples employing an optimized luminophore mix in conjunction with visibly-transparent, spectrally-selective diffraction gratings.

**Construction of industry-ready up-scaled energy-generating window prototypes**

We constructed an up-scaled photovoltaic window prototype using 16 of the 100mm×100mm 1D diffraction gratings of period 20 μm, placed around the glass perimeter region. A double-luminophore-layer, similar to that of Sample #4 (described in Table 1 of the main manuscript), was used. 27 mm-wide CIS solar cell cut-outs were used at sample edges, and additionally we installed 27mm-wide backside-mounted cell modules glued onto the perimeter of glass structure, to enhance the energy-harvesting performance by fully utilising the light concentration effects produced near the sample edges. This performance enhancement may be necessary for achieving net-zero power balance in future buildings, since in the presence of any light concentration effects, the near-edge glass panel regions also exhibit the strongest light losses, a part of which can be recovered in this way.

Figure S5 shows this 500mm × 500mm microstructured glass concentrator sample powering several ventilation fans in outdoor illumination conditions in autumn. The framing was provided by DuoGlass (Joondalup, Western Australia) using industry-standard Kӧmmerling plastic framing system.


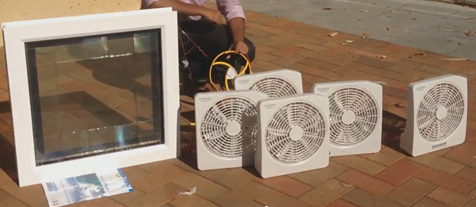


**Figure S5.** **Powering household appliances.** A 500mm x 500mm framed microstructured-glass concentrator during outdoor performance testing in May 2015. All five of the series-connected DC fan systems were able to run simultaneously at an appreciable rotation rate when powered by the sample.

During sample testing on May 07, 2015 at 12:30 pm, the following electric output parameters were measured: V_oc_ = 39.2 V and I_sc_ = 210 mA. The I-V curve data was measured on a later date, resulting in the module fill factor of FF = 0.525. Using these data, we can obtain a 4.32 W figure for the maximum electric power output actually observed from this sample so far in field conditions, with the CIS cell areas being at their working temperature near 48-50 °C.

Out of the 210 mA measured, the separately-measured I_sc_ output contribution of the backside perimeter-mounted cells was about 115 mA.

The development of improved-performance, commercial-ready framed photovoltaic windows of size 500mm × 1000mm and larger is ongoing at Edith Cowan University (ECU) and ClearVue Pty Ltd (www.clearvuepv.com).

**Near-infrared fluorescence quantum yield measurements and data**

Rare-earth doped ceramics known to emit efficient fluorescence in the near-IR wavelength region by near-IR excitation attract great interest in a range of fields, from applied photovoltaics to bioimaging applications. Absolute fluorescence quantum yield (QY) measurements of a powder sample of Y_2_O_3_ sensitised with Yb and codoped with several rare-earth ions (luminophore *α* as per material description first reported in Ref. 16 of the main manuscript) have been performed at Edinburgh Instruments Ltd. using a double grating FLS920 spectrometer. Powder sample was placed in-between two thin quartz slides, which were clamped into a standard front face samples holder; Edinburgh Instruments integrating sphere was used for QY measurements, and Benflect plug was used as a reference sample. A continuous xenon lamp (Xe1, 450W) was used for excitation. An NIR-PMT (Hamamatsu R5509) detector was used for photon detection. The sample was excited at near 905 nm and signal was measured at 0.5 nm step for scattering peaks and 1 nm for emission range with 2 s integration time. The slits were 10 nm on excitation and 3.5 nm on emission arms. At such wide slit widths the scattering signal was very large, and could not be measured directly, therefore a neutral density filter of 10% transmissivity was used in the excitation path for scanning over scattering peak. For QY calculation, the scattering peaks were scaled up by factor 10. The ratio of the number of emitted photons to the number of absorbed photons was quantified using the difference of areas under the Rayleigh scattering peaks of the reference and the phosphor sample (Fig. S6(a)), and the difference of areas under the spectrally corrected emission peak of the sample and background signal (reference), as shown in Fig. S6(b). The calculations were performed by the QY Wizard of F980 software. The results showed QY = (6.9 ± 0.4)% for the entire emissions range between 927 nm and 1625 nm. Experimental data and details are summarised in Figure S6.


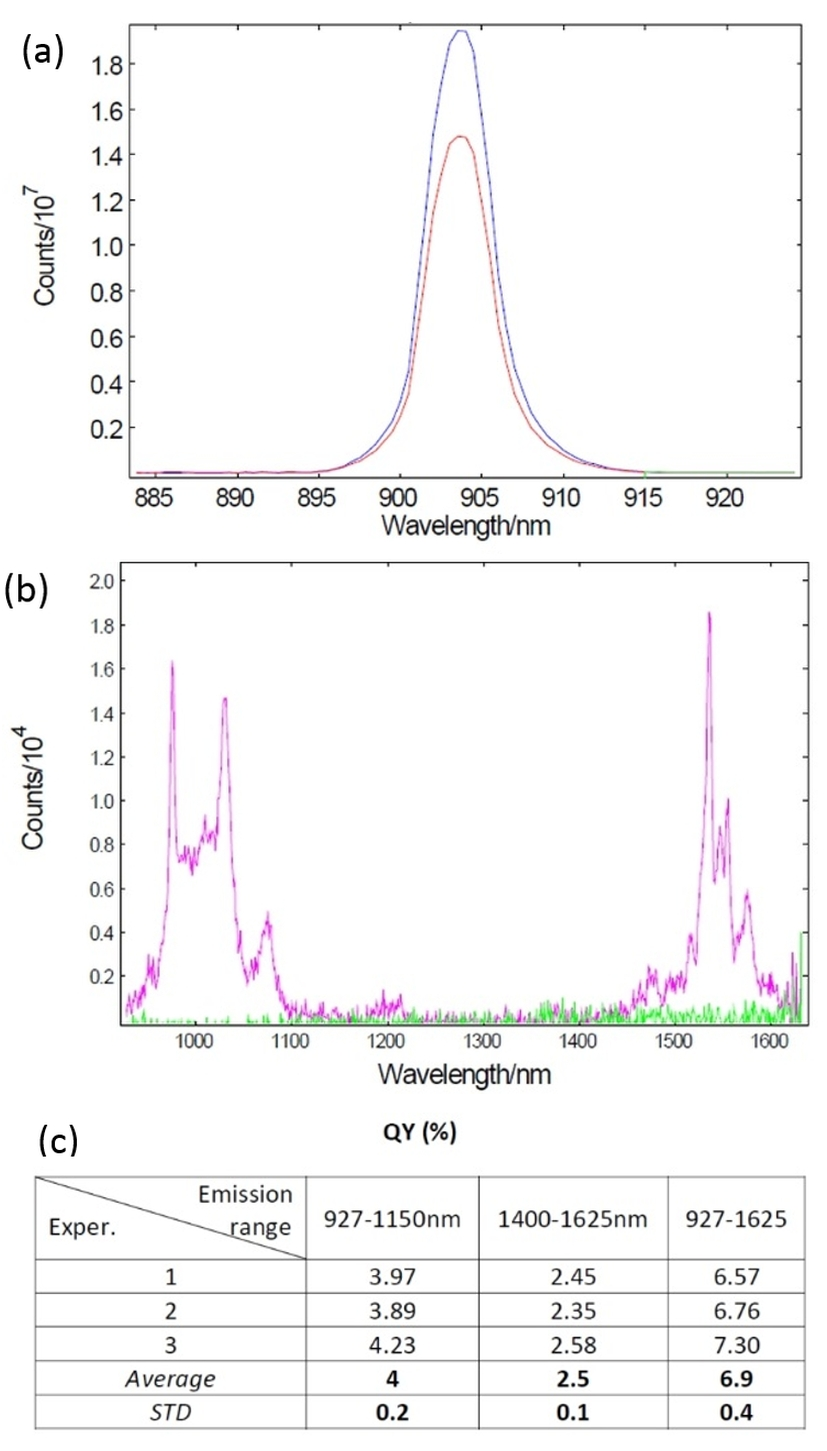


**Figure S6.** **Absolute fluorescence quantum yield measurement results obtained from Y_2_O_3_-based phosphor material.** (a) Scattering spikes of the reference (blue trace) and the sample (red trace); (b) emissions of sample (pink trace) and background signal (green trace); (c) experimental datasets for the absolute fluorescence quantum yield measured in different spectral ranges of emission, obtained for excitation near 905 nm. Data and measurements are courtesy of Edinburgh Instruments Ltd., Scotland, UK.

While the measured QY values of luminophore *α* are moderate ((4±0.2)% for emissions in the range between 927 and 1150 nm), this is still a remarkable fluorescence quantum yield result for any inorganic powder phosphor material excitable in a broad near-IR range between 900-1000 nm. In particular, this QY value exceeds all fluorescence quantum yields reported in Ref. 18 of the main manuscript for several organic dye-based materials capable of near-IR luminescence excitation.

The second luminophore material used in this study (inorganic powder of composition type ZnS:(Ag, Tm)) was a commercial luminophore possessing very large Stokes shift, emitting a narrow peak near 795 nm when excited within a broad absorption band between 300-430 nm. The fluorescence quantum yield of ZnS activated with Ag has been reported to exceed 40% in the long-wavelength UV range and to reach 120% for short-wavelength UV excitation (Ref. 19 of the main manuscript).
